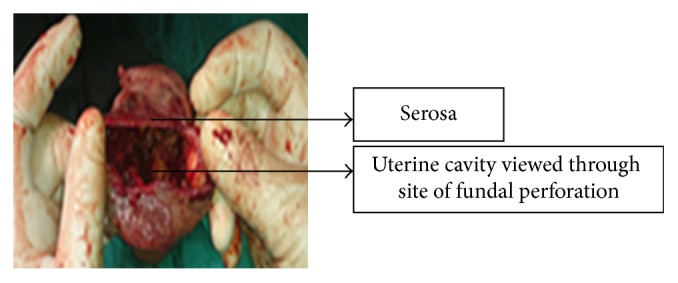# Placenta Percreta in a Gravid Bicornuate Unicollis Uterus

**DOI:** 10.1155/2017/4082182

**Published:** 2017-06-11

**Authors:** Sammy Ngichabe, Mandeep Sura

**Affiliations:** ^1^Nairobi Women's Hospital, Nairobi, Kenya; ^2^University of Nairobi, Nairobi, Kenya

## Abstract

**Background:**

Identifying bicornuate uterus can be challenging especially as a cause of early pregnancy bleeding. On ultrasonographic examination, it is difficult to misdiagnose pregnancy in a bicornuate uterus as an ectopic pregnancy due to the continuity of the endometrium. A rudimentary horn of a bicornuate uterus in early pregnancy can occasionally be misdiagnosed for an ectopic pregnancy especially when compounded by severe abdominal pains and supportive sonographic evidence. Myometrial invasive grading of placenta may be necessary for emergency preparedness and consenting. Hemihysterectomy is lifesaving when percreta has caused severe postpartum haemorrhage.

**Case Presentation:**

We present a 24-year-old primigravida who presented to the maternity department with severe abdominal pains at 35 weeks. She was pale on clinical examination and haemodynamically unstable. She underwent emergency caesarean section with a preoperative diagnosis of concealed abruptio placentae. Intraoperatively we encountered a bicornuate uterus, delivered a fresh stillbirth, and noted a placenta percreta. A hemihysterectomy was done and she recovered after transfusion without complications.

**Conclusion:**

A gravid horn of a bicornuate uterus may present as an ectopic pregnancy; careful assessment at laparotomy or laparoscopy is required to prevent inadvertent surgical termination of pregnancy. Placental myometrial invasive assessment is important for delivery emergency preparedness.

## 1. Background

Overall, congenital Müllerian anomalies occur in ~1.5% of females (0.1–3%) [1], with bicornuate uterus constituting 25% of Müllerian class 4 uterine anomalies [2]. Grimbizis et al. reported a prevalence of 4.3% for the general population, about 3.5% in infertile women, and about 13% in women with recurrent pregnancy losses [3].

**Figure 1:**
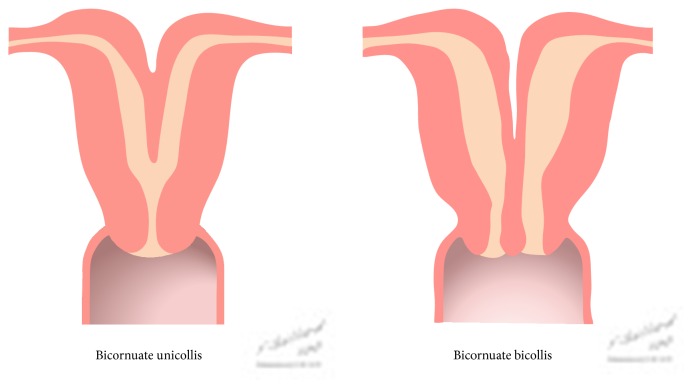


The pathophysiology of this type of anomaly involves incomplete fusion of both uterine horns during embryogenesis; the bicornuate uterus is formed when the Müllerian ducts incompletely fuse at the level of the uterine fundus. In this anomaly, the lower uterus and cervix are completely fused, resulting in 2 separate but communicating endometrial cavities, a single-chamber cervix and vagina. A muscular intrauterine septum is also present, and this defect corresponds externally to an indentation or groove at the fundus. Subclassification into complete or partial categories depends on septum length. Complete uterine septa that extend either to the internal or external os are known as bicornuate unicollis uterus and bicornuate bicollis uterus, respectively (Figure 1) [4].

## 2. Case Report

We present a rare case of Müllerian dysgenesis of P.C., a 24-year-old primigravida who presented to the maternity emergency department with severe generalised abdominal pains at 35 weeks. She was pale on clinical examination and haemodynamically unstable.

Abdominal palpation revealed a 35 cm fundal height, longitudinal lie, and baby in cephalic presentation; her abdomen was too tender for deeper palpation; fetal heart tones could not be heard on hand held Doppler and cardiotocographic auscultation. No bleeding was noted vaginally with a closed cervical os.

She had a subumbilical midline scar which on further inquiry was due to what was a negative laparotomy for preoperative diagnosis of ectopic pregnancy at 8-week gestation during the current pregnancy. No abnormalities were noted during surgery at that point.

She was resuscitated with two litres of normal saline and six units of blood grouped and cross matched. Emergency caesarean section was done with a tentative preoperative diagnosis of concealed abruptio placentae.

**Figure 2:**
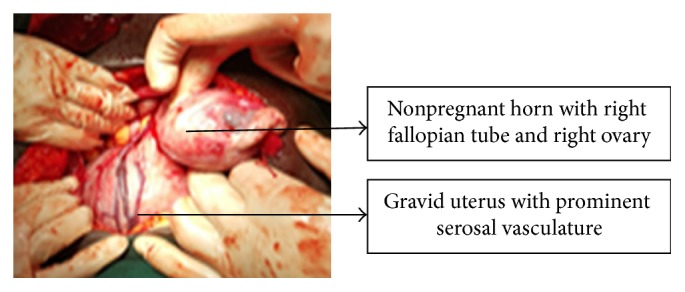


Abdominal entry through a subumbilical midline incision was employed; two litres of haemoperitoneum and tortuous vessels over the gravid uterus were encountered; on further exploration bicornuate uterus was noted intraoperatively (Figure 2).

The traditional Kerr uterine incision was made and baby delivered (fresh stillbirth). Placental tissue was adherent to entire myometrium all the way to serosa, with areas of active haemorrhage at point of attachment. Attempted removal left a large defect that was actively bleeding (Figure 3).

Upon further evaluation a decision to perform unilateral caesarean hemihysterectomy was made due to active on going haemorrhage at the point of placental attachment.

Bilateral ovarian preservation was carried out successfully; the uterine vessels on the ipsilateral side were skeletonized and ligated achieving haemostasis. The ureters were identified from the level of the pelvic brim and followed along their entire length to the bladder to rule out any possible urinary tract abnormalities that occasionally coexist with Müllerian anomalies.

Lavage was done and abdomen closed in layers. She received 4 units of whole blood and recovered without complications.

## 3. Discussion

This rare occurrence is presented with most of the possible acute emergencies in obstetrics right from early pregnancy to delivery. Bicornuate uterus may not be the commonest differential diagnosis one would be looking for in a gynaecologic clinic especially when there has not been any antecedent infertility, miscarriage, menstrual irregularity, or abnormal uterine bleeding history as in our case.

Bicornuate uterus may present with menstrual abnormalities or miscarriages or may be asymptomatic only to be diagnosed retrospectively. This may be during management for miscarriages, caesarean delivery, or hysteroscopy or following hysterosalpingography for fertility workup [5]. It may rarely be misdiagnosed for ectopic pregnancy as the pregnant horn may sonographically resemble an ectopic with the attendant nonpregnant uterus. Judicious approach at laparotomy and laparoscopy is required during surgical intervention for suspected ectopic pregnancies. Possible risks during surgical intervention may include inadvertent hemihysterectomy for a pregnancy that would otherwise have grown to viability [6]. This patient had a negative laparotomy for ectopic pregnancy and was fortunate the pregnancy was not interfered with as both tubes were grossly normal with no intraperitoneal haemorrhage noted. She was reviewed at 20 weeks with a scan showing a live intrauterine pregnancy and hence followed up as per the normal routine pregnancy schedule.

She was admitted in late third trimester with severe abdominal pains and was haemodynamically unstable necessitating emergency caesarean section where a placenta percreta was encountered. There is no strong evidence for placental assessment screening due to paucity of cases to conduct large studies; however there are cases of placental accreta and hemihysterectomy encountered intraoperatively in bicornuate uterus [7]. A colour flow Doppler ultrasound or MRI of placenta would be helpful in establishing whether abnormal placentation exists for better planning of delivery and consenting processes whereby hemihysterectomy is discussed prior to delivery.

## 4. Conclusions

A gravid horn of a bicornuate uterus may rarely be misdiagnosed for an ectopic pregnancy in early pregnancy; however endometrial lining continuity is of paramount to assess and rule out other forms of Müllerian dysgenesis sonographically. Postpartum haemorrhage due to placenta percreta is ultimately managed by caesarean hysterectomy.

## Figures and Tables

**Figure 3:**